# Hillock Related Degradation Mechanism for AlGaN-Based UVC LEDs

**DOI:** 10.3390/nano13091562

**Published:** 2023-05-06

**Authors:** Juntong Chen, Jianxun Liu, Yingnan Huang, Ruisen Liu, Yayu Dai, Leming Tang, Zheng Chen, Xiujian Sun, Chenshu Liu, Shuming Zhang, Qian Sun, Meixin Feng, Qiming Xu, Hui Yang

**Affiliations:** 1 School of Nano Technology and Nano Bionics, University of Science and Technology of China, Hefei 230026, China; chenjuntong2021@sinano.ac.cn (J.C.); ynhuang2018@sinano.ac.cn (Y.H.); yydai2020@sinano.ac.cn (Y.D.); xjsun2018@sinano.ac.cn (X.S.); csliu2022@sinano.ac.cn (C.L.); mxfeng2011@sinano.ac.cn (M.F.); hyang2006@sinano.ac.cn (H.Y.); 2 Key Laboratory of Nanodevices and Applications, Suzhou Institute of Nano-Tech and Nano-Bionics, Chinese Academy of Sciences, Suzhou 215123, China; 3 Guangdong Institute of Semiconductor Micro-Nano Manufacturing Technology, Foshan 528000, China; rsliu2021@sinanogd.ac.cn (R.L.); lmtang2021@sinanogd.ac.cn (L.T.); zchen2022@sinanogd.ac.cn (Z.C.); 4 GuSu Laboratory of Materials, Suzhou 215123, China; xuqiming2021@gusulab.ac.cn

**Keywords:** UVC lighting emitting diodes, aluminum gallium nitride, reliability, hillock, threading dislocations, current leakage

## Abstract

Heteroepitaxial growth of high Al-content AlGaN often results in a high density of threading dislocations and surface hexagonal hillocks, which degrade the performance and reliability of AlGaN-based UVC light emitting diodes (LEDs). In this study, the degradation mechanism and impurity/defect behavior of UVC LEDs in relation to the hexagonal hillocks have been studied in detail. It was found that the early degradation of UVC LEDs is primarily caused by electron leakage. The prominent contribution of the hillock edges to the electron leakage is unambiguously evidenced by the transmission electron microscopy measurements, time-of-flight secondary ion mass spectrometry, and conductive atomic force microscopy. Dislocations bunching and segregation of impurities, including C, O, and Si, at the hillock edges are clearly observed, which facilitate the trap-assisted carrier tunneling in the multiple quantum wells and subsequent recombination in the p-AlGaN. This work sheds light on one possible degradation mechanism of AlGaN-based UVC LEDs.

## 1. Introduction

Over the past few years, AlGaN-based ultraviolet light-emitting diodes (LEDs) emitting between 260 nm and 280 nm (near-UVC) have the potential to supersede conventional mercury lamps in sterilization and disinfection fields, due to their various advantages of miniaturization, short switch times, low operation power, and environmental friendliness [1,2,3,4]. Especially, the worldwide spread of the SARS-CoV-2 virus has further raised demands for improving the performance and reliability of UVC LEDs to boost market penetration [5].

However, so far, the lifetime of UVC LEDs can only reach a few thousand hours [1,6]. The degradation of UVC LEDs is closely attributed to a high density of threading dislocations (TDs) [7,8], the generation, and the diffusion of point defects [9,10]. In fact, since large lattice mismatches and thermal-expansion-coefficient mismatches between the AlGaN films and the substrate typically lead a stress accumulation [11], the formation of massive micron-scale hillocks on the AlGaN surface for strain relaxation has been proposed as an important origin in device degradation [12,13]. The rough hexagonal hillock defects and resultant in-plane phase separation due to weak surface migration of Al atoms on the surface have been demonstrated to degrade the luminescence of AlGaN multiple quantum wells (MQWs) and monochromaticity of the LEDs [14,15]. It has also been confirmed that the local overheating caused by the current leakage around the hillock led to an abrupt catastrophic degradation of the LEDs after a long-time constant current stress [12]. Furthermore, the growth kinetics of the hillocks is believed to affect the migration of both TDs and point defects, which also impacts the incorporation of impurity adatoms around the hillocks [16,17], further enhancing the non-radiative recombination component and deep-level luminescence, which then decreases the quantum efficiency of the UVC LEDs. Nevertheless, these hillock-related degradation mechanisms for the UVC LEDs remain unresolved.

In this work, the impurity/defect behavior and degradation mechanism of the UVC LEDs, in close relation to the hexagonal hillocks, have been intensively investigated. The significant optical degradation of UVC LEDs during the early stage of aging is found to be strongly related to electron leakage. The electroluminescence (EL), current–voltage (I–V) measurement, transmission electron microscopy (TEM), time-of-flight secondary ion mass spectrometry (Tof-SIMS), and conductive atomic force microscopy (C-AFM) results reveal that the hillock edges contribute most to the electron leakage, which involves substantial dislocations bunching and impurity segregation at the hillock edges.

## 2. Experiment

**Figure 1:**
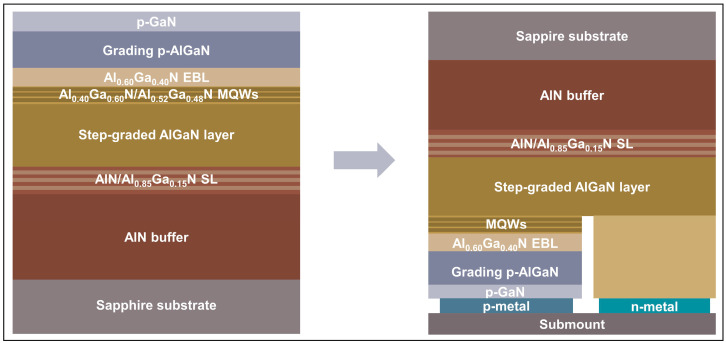
The schematic production procedure of the UVC LED sample.

The UVC-LED samples were grown on a *c*-plane sapphire substrate by metal-organic chemical vapor deposition starting with a 4 μm thick AlN buffer layer, followed by 300 nm thick AlN/Al_0.85_Ga_0.15_N superlattice (SL) transition layers, and then, 3 μm thick AlGaN films with step-graded Al mole fractions from 0.75 to 0.5. The active region consisted of five-loop Al_0.4_Ga_0.6_N/Al_0.52_Ga_0.48_N MQWs. The *p*-side comprised a 25 nm thick Mg-doped Al_0.6_Ga_0.4_N electron blocking layer (EBL), a 50 nm thick p-Al_x_Ga_1−x_N (*x* from 0.4 to 0) grading layer, and a 10 nm thick p-GaN contact layer on the top. After epitaxial growth and chip-processing, the UVC LED clips were flip-chipped mounted at a size of 500 × 250 μm^2^. The schematic production procedure of the UVC LED sample is displayed in Figure 1. In order to investigate the degradation behavior during accelerated aging of the LEDs, the devices were submitted under a constant current of 100 mA (current density ~190 A/cm^2^) at room temperature for 4 h. To explore the impurity/defect behavior associated with hillocks, a 400 nm thick Si-doped n-Al_0.43_Ga_0.57_N film was grown separately on a similar 3 μm thick AlGaN template.

The cathodoluminescence (CL) mappings, in combination with scan electron microscopy (SEM), was carried out by FEI Quanta 400 FEG with a Gatan MonoCL3+ system at room temperature, under an acceleration voltage of 10 kV. The EL spectra of the LEDs were collected by an MPI CORPORATION U-P2 system with an integrating sphere. The electrical degradation of the LEDs was investigated by a KEITHLEY semiconductor parameter analyzer. The micro-Raman scattering was carried out by LabRAM HR in a backscattering geometry and $Z(\mathrm{XX})\overset{-}{Z}$ configuration at room temperature, with a 532 nm Ar^+^ laser beam as an excitation source focusing on the (0001) surface of n-AlGaN sample, which keeps the incident and back-scattered polarization photons parallel. The charge-coupled-device camera was used for recording the Raman spectrum by detecting the E2 (GaN-like) and A1(LO) photon modes. The cross-sectional bright-field TEM images were measured with a 200-kilovolt Talos F200X STEM.

The impurity depth profiles of C, O, and Si of n-AlGaN sample were detected by ION Tof.SIMS5-100 system. For the Tof-SIMS measurement, Cs^+^ incident ions were applied to sputter the surface of the n-AlGaN sample at 45° and a Bi^+^ liquid metal gun was used for the analysis. The sputtering rate was equipped with 82 nm/h and the sputtering depth almost penetrated the n-AlGaN layer on the top. The depth and lateral resolution were less than 1 nm and 100 nm, respectively. The morphology and corresponding current images of the n-AlGaN sample were acquired by operating Dimension ICON AFM in contact mode with a Pt-coated tip and tunneling amplifier module. We deposited a Ti/Al/Ti/Au electrode on the sample surface, which was connected with the metal substrate using a silver paste, to form the Ohmic contact. It was interfaced with the tip-to-sample Schottky contact to compose an external current circuit. The tip scanned over one hillock structure at a rate of 0.5 Hz, and a forward 5 V bias, which meant a negative sample-to-tip voltage (−5 V) for the n-type sample was applied across the hillock. The current image was recorded by tunneling the amplifier module.

## 3. Results and Discussion

### 3.1. Carrier Overflow in the Initial UVC LED

**Figure 2:**
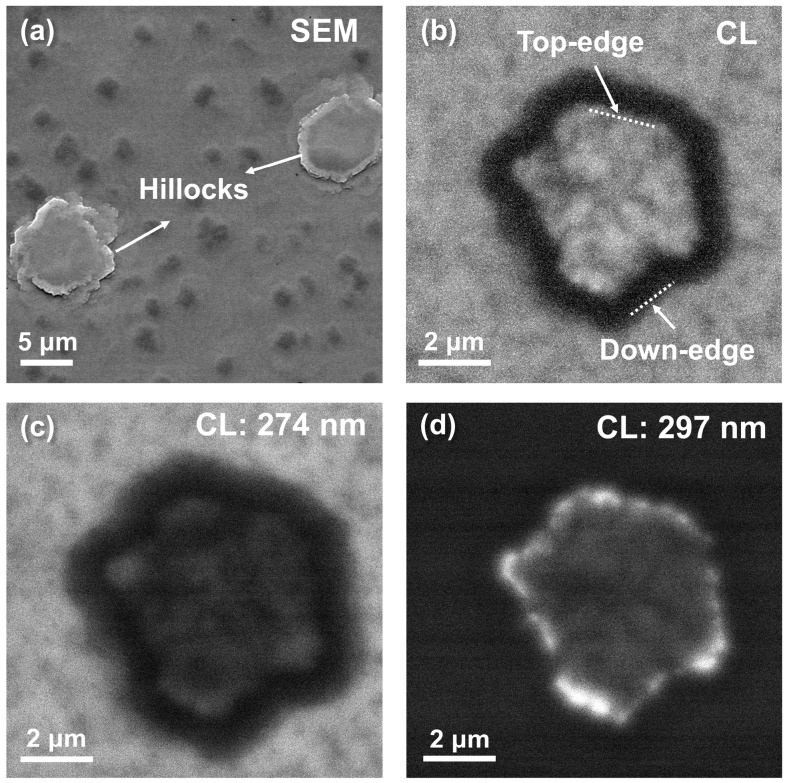
(**a**) SEM image of the p-side surface of the initial UVC LED surface. (**b**) Room temperature panchromatic CL image of one representative hillock under an acceleration voltage of 10 kV. Monochromatic spatially resolved CL images of the same hillock area were acquired at (**c**) 274 nm and (**d**) 297 nm.

Figure 2a shows the SEM image of the p-side surface of the initial UVC LED, from which several hillocks with a diagonal ranging from submicron to several micrometers can be observed. The density of hillocks on the surface is ~1.6 × 10^5^ cm^−2^. These surface hillock defects are caused by the accumulated compressive strain during the AlGaN growth and also the spiral growth induced by TDs [17]. Figure 2b gives the panchromatic CL image of one representative hillock. The hillock down-edges are dark in the CL images, indicating the presence of high-density point defects or dislocations that act as non-radiative recombination centers. Monochromatic spatially resolved CL images were further taken to observe the emission distribution around the hillock at wavelengths 274 and 297 nm. As shown in Figure 2c,d, the monochromatic CL maps obtained at the lower energy (297 nm) and higher energy (274 nm) are almost complementary, providing the evidence that the in-plane phase separation exists in AlGaN, with the hillock top-edge and the hillock-free area showing a non-uniform light emission and composition inhomogeneity.

**Figure 3:**
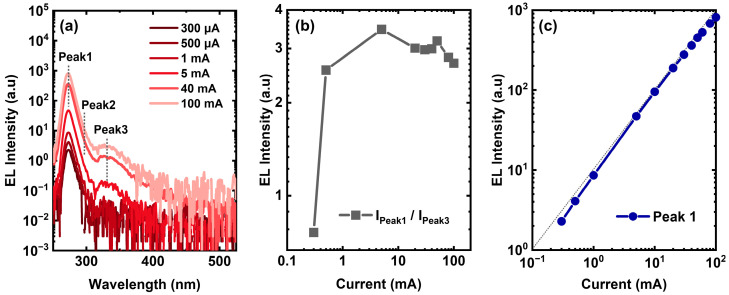
(**a**) EL spectra of the initial UVC LED at different injected currents. (**b**) The intensity ratio between the dominant emission (Peak1) and lower energy emission (Peak3). (**c**) Correlation between the emission intensity of Peak1 and injected current.

As shown in Figure 3a, the electroluminescence (EL) measurements of the initial UVC LED were carried out at different injected currents. The emission peak between 250 nm and 300 nm shows an asymmetry, which can be identified as a dominant emission Peak1 at 274 nm and a lower energy emission Peak2 at around 297 nm. In combination with the CL results in Figure 2c,d, Peak1 in the EL spectra originates from the native luminescence in the hillock-free areas, while Peak2 corresponds well to the intrinsic emission at the hillock top edges. Moreover, it was noticed that a broad emission band (Peak3) centered around 330 nm gradually appears as the current increases. Several physical processes might be involved in the emission of Peak3: (i) the deep-level recombination in the active region [18], (ii) the field-enhanced carriers escaping to the last barrier, and then, deep-level recombination [19], (iii) the carriers overshoot across the EBL to p-AlGaN layers, and then, recombination [20], (iv) the trap-assisted tunneling (TAT) of carriers into the EBL or p-AlGaN, and the subsequent deep-level transitions [10,21,22].

A plausible candidate for the origin of Peak3 is the overflow of the carriers from the MQWs towards the p-side, due to its presence only at higher injected currents, which can also be demonstrated by the variation in the intensity ratio between Peak1 and Peak3 (I_Peak1/_I_Peak2_), as the injected current increases. As shown in Figure 3b, the intensity ratio increases at first, then, tends to saturate at 5 mA and subsequently decreases, verifying a growing trend in the electron-escaping probability from the active region at higher current levels. In fact, as shown in Figure 3c, the quasi-linear dependence of the dominant emission (Peak1, 274 nm) on the injected current has clearly evidenced that the classical band-to-band transition dominates the emission of Peak1 [23]. Therefore, Peak3 at high injected currents is considered to result from the carriers overflow rather than from the deep-level recombination in the active region, as mentioned in processes (i) and (ii) above.

### 3.2. Electron Leakage in the Aged UVC LED

**Figure 4:**
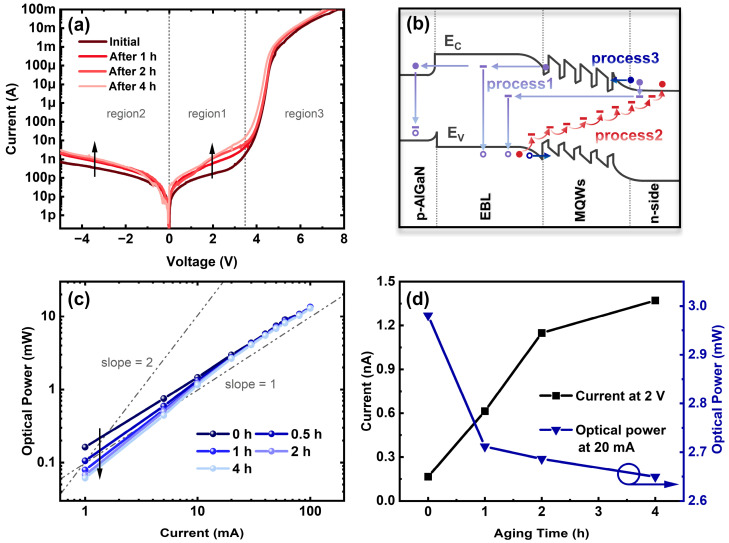
(**a**) Logarithmic scale forward I–V characteristics of the initial and aged UVC LEDs. (**b**) Schematic band diagram with various carrier transport processes explaining the change in I–V results. (**c**) Optical power versus injected current (L–I) curves in the initial and aged UVC LEDs. (**d**) Dependence of the forward subthreshold current and optical power on aging time.

To further clarify whether the overflow of the carriers was brought by weak barrier confinement (iii) or TAT (iv), the current–voltage (I–V) measurements of the initial and aged UVC LEDs were performed. As shown in Figure 4a, three current regions can be identified in the I–V curves: region1—the forward bias region below the threshold voltage (from 0 V to 3.5 V); region2—the reverse bias region; region3—the high-forward bias region (above 3.5 V). It is obvious that both the reverse leakage and forward subthreshold current remarkably increase after stressing for 4 h under a constant current of 100 mA.

We further established the corresponding physical models for the three regions above, as shown in Figure 3b. For region1, the forward subthreshold current was typically caused by TAT, that the traps in the MQWs or EBL could assist electrons from escaping towards the p-side, which can be modeled as the superposition of an ideal diode and a parallel resistor [24] (process1 in Figure 4b). We found in Figure 4a that the forward subthreshold current already existed before stress, indicating the presence of TAT-related defects within the as-grown AlGaN MQWs. Therefore, it can be inferred that trap-related electron leakage was the main cause of the parasitic emission of Peak3 in Figure 3a. The increase in the forward subthreshold current after stress is mostly related to the activation of these as-grown defects. With the L–I curves measured on the UVC LEDs during stress (Figure 4c), Figure 4d shows the dependence of the forward subthreshold current and optical power of the LED on the aging time. With increasing aging time, the subthreshold current rapidly increases, while the optical power dramatically decreases. Thus, it can be concluded that the significant degradation of UVC LEDs in the early stage of aging is primarily caused by electron leakage.

However, as shown in Figure 4c, it is noteworthy that the optical power decreases more significantly and the slope of the logarithmic L–I curve changes from 1 towards 2 at the lower injected currents (<10 mA) during stress. This indicates that the stress leads to a rise of non-radiative recombination centers, with a resultant rise in the non-radiative recombination component [25,26]. Thus, the optical degradation is not only related to the electron leakage but also involved in the non-radiative recombination. The enhanced non-radiative recombination is probably associated with the point defects in the active region, which will induce an increase in the reverse leakage current, as exhibited in Figure 4a. Process2, in Figure 4b, clarifies the dominant reverse parasitic leakage paths, with trapped electrons transitioning from the p-side valence band to the n-side conduction band by the thermal activation and multistep tunneling through deep-level states [27]. Additionally, process3 (Figure 4b) corresponds to the diffusion-recombination current under the thermionic field in a high-forward bias (region3 in Figure 4a). The slight drop in driving voltage after stress is attributed to the decrease in the p-side contact resistivity [28] or the activation of Mg acceptors in p-AlGaN [10].

**Figure 5:**
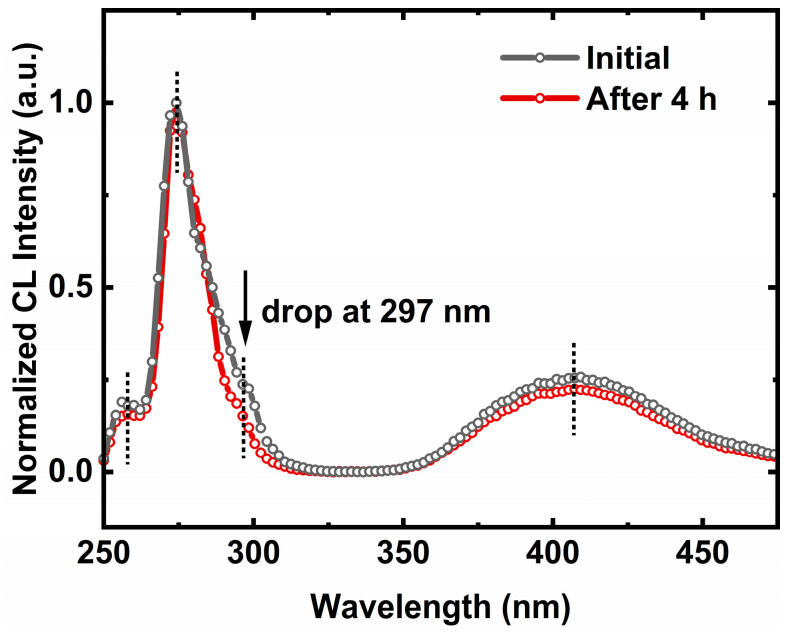
Normalized integrated CL spectra of the initial and aged UVC LEDs were obtained under an acceleration voltage of 10 kV.

To study the origin of the electron leakage, we compared the normalized integrated CL spectra in the hillock area on the UVC LEDs before and after stress. As shown in Figure 5, the weak parasitic peak at 260 nm in the CL spectra originated from the band-to-band transition of the underlying n-AlGaN. The dominant emission includes the main peak at 274 nm and a weak shoulder at 297 nm, corresponding to the emission from the hillock-free area and hillock top-edges, respectively. The additional broad emission band at 410 nm typically originates from the deep-level recombination, which is involved in ${\left(V_{Ⅲ}\right)}^{3-}$ and $V_{Ⅲ}$ complex related transitions of n-AlGaN [29]. A careful comparison between the CL spectra and EL spectra (Figure 3a) revealed the absence of the emission centered around 330 nm in the CL spectra. This also illustrates that the parasitic emission at around 330 nm in the EL spectra is mainly attributed to the electron leakage into the p-side, under the electric field, since CL excitation only occurs along the penetration path with the emission intensity closely related to the layer thickness. Moreover, it is interesting that the near-band emission intensity of the CL spectra remains stationary after stress, while the intensity of the shoulder at 297 nm exhibits an obvious drop. Since the lower energy emission of 297 nm is from the hillock top edges, as shown in Figure 2d, it is considered that the electron leakage is closely related to the hillock top edges.

### 3.3. Electron Leakage at the Hillock Edge

**Figure 6:**
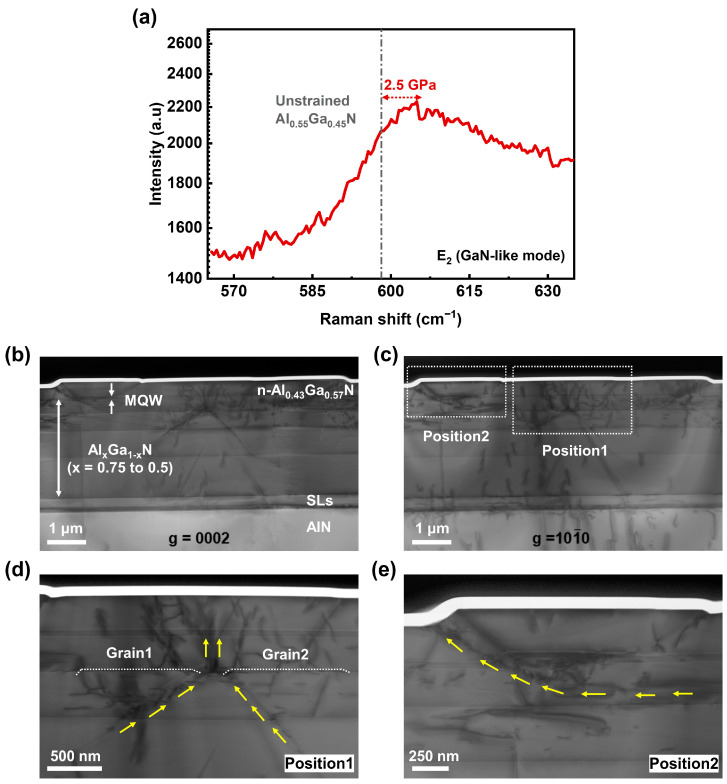
(**a**) Raman spectrum of the n-AlGaN sample. Cross-sectional TEM images of a hillock in the n-AlGaN sample taken with the diffraction vector (**b**) g=0002  and (**c**) $g=10\overset{-}{1}0$. Cross-sectional TEM images of (**d**) position1 and (**e**) position2 showing the evolution of hillock and dislocations.

In order to investigate the contribution of the hillock edges to the electron leakage, micro-Raman scattering, TEM, Tof-SIMS, and C-AFM measurements were performed on samples with n-AlGaN on top. As plotted in Figure 6a, the Raman spectrum of photons of the n-AlGaN sample displays the E_2_ (GaN-like) mode at about 604.9 cm^−1^. It has been reported that the phonon frequency of unstrained thick Al_0.55_Ga_0.45_N (average Al content of our sample) was located at 598.5 cm^−1^ [30]. The relationship between the E_2_ (GaN-like) phonon frequency shift ∆ω and the effect of biaxial stress $\sigma_{\mathrm{xx}}$ can be formulated as follows [31]:

(1) $$\sigma_{\mathrm{xx}}=\frac{∆\omega }{2.56}$$

In contrast to the unstrained Al_0.55_Ga_0.45_N layer, the frequency right shift of our sample indicates the existence of a compressive stress of 2.5 GPa in the epitaxial layers.

Such a large total compressive stress has been partly released by the generation of hillocks and dislocations. Figure 6b,c show the bright-field TEM images of one hillock with g=0002  and $g=10\overset{-}{1}0$, respectively, from which dislocations with Burgers vectors of type a and (a + c) are mainly distributed in AlGaN layers. It can be seen that the AlGaN layer on the SL transition layers presents a sharp interface in the pre-growth stage, yet then, roughens by forming hillock grains as the total strain energy increases with thickness. As shown in position1 (Figure 6d), grain1 and grain2 have been gradually generated to relax the compressive strain. As the grain grows, the dislocations would bend towards the side facet of the grain (see yellow arrows), leading to substantial TDs bunching at the step edges. It is noteworthy that the grain coalescence has produced a larger hillock, the center of which corresponds to the grain boundary and generates a large number of fresh TDs due to the inherent twist or tilt of adjacent grains [11]. These newly formed TDs that are the channel of strain relaxation will cause leakages and finally affect the reliability of the device. As shown in position2 in Figure 6e, the further growth of the hillock would also drive the TDs bending and bunching towards the side facet due to the mirror force, which is consistent with the area of the lower luminescence and higher leakage.

**Figure 7:**
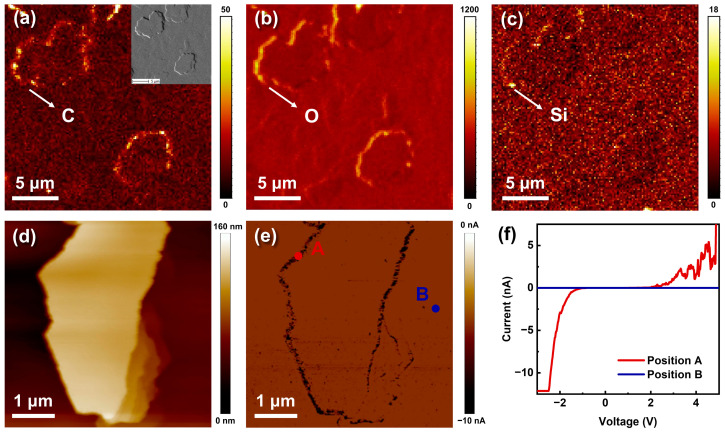
Tof-SIMS mappings of C (**a**), O (**b**), and Si (**c**) over hillocks of the n-AlGaN sample. The inset in (**a**) shows the SEM image of the analyzed region. (**d**) Surface morphology and (**e**) C-AFM current mapping of the hillock at sample-to-tip voltage of −5 V. (**f**) Local I–V curves at position A and position B in (**e**).

The impurities incorporation of C, O, and Si is also found to correlate very well with the hillock. As shown in the Tof-SIMS mappings in Figure 7a–c, impurities are found to segregate around the hillock edges. On the one hand, the strain field of TDs that bunch at the hillock edges can promote impurities to incorporate around [16]. On the other hand, the impurity incorporation should be kinetically favored at the hillock edges due to their abundant dangling bonds [32]. Moreover, the edge-type dislocations that are the primary dislocations bunching at the hillock edges can also provide additional dangling bonds [33]. Therefore, it can be presumed that more point defects are introduced at the hillock edges, locally favoring the formation of parasitic leakage paths.

The C-AFM was employed to further investigate the role of impurity segregation on the local electrical property. The surface morphology and corresponding current mappings of the hillock in a 5 V forward bias are displayed in Figure 7d and e, respectively. Under forward bias, the dark region in the current mapping proves higher current conduction at the hillock edges. To avoid the influence of topographic contrast on the real current signals, the local I–V curves were taken from position A at the hillock edge and position B in the hillock-free area. As shown in Figure 7f, position A conducts rapidly at a forward bias higher than 1 V and shows an obvious leakage current at reverse bias, while position B has poor electrical conductibility, further evidencing that substantial conductive and leakage centers exist at the hillock edges. These high conductive and leakage centers are considered to be related to the impurities and TDs at the hillock edges, which lead to a rise in trap states in the MQWs and EBL, effectively enhancing the TAT process at the hillock edges. During the early aging of UVC LEDs, the activation of these point defects promotes an increased electron leakage, explaining the rise in the forward subthreshold current (Figure 4a) and the optical degradation of the hillock top edges (Figure 5).

In fact, several groups have reported the transition mechanisms for the parasitic emission centered around 3.7 eV in AlGaN, including the conducting band to Mg-related deep levels [22], and $V_{N}^{3+}$ to neutral Mg-acceptors [34] or valance band [35]. Since the bandgap energy of the Al_0.60_Ga_0.40_N EBL and p-Al_0.40_Ga_0.60_N layer is 4.84 eV and 4.28 eV, respectively, considering the binding energy of $V_{N}^{3+}$ state in Al_0.60_Ga_0.40_N and Al_0.40_Ga_0.60_N is around 0.7 eV and 0.6 eV [36], respectively, while the binding energy of Mg-related acceptors is 0.3 eV–0.4 eV in Al_x_Ga_1−x_N (x from 0.27 to 0.7) [34], we can speculate that the luminescence around 3.7 eV in this work is mainly from the $V_{N}^{3+}$ to the valance band transition in the p-AlGaN.

## 4. Conclusions

In conclusion, the hillock-related degradation mechanism of 274 nm in UVC LEDs was investigated. It revealed that huge compressive stress can be the driving force for the hillock generation, and further growth of the hillocks will promote the TDs bending and bunching towards the side facet due to the mirror force. The impurity segregation of C, O, and Si around the hillock edges was verified by the Tof-SIMS mappings. Both the TDs bunching and impurity segregation at the hillock edges that permeate the MQWs and EBL region can act as the primary leakage paths by enhancing the trap-assisted-tunneling and subsequent radiative recombination in the p-AlGaN, leading to the optical degradation of the UVC LEDs. This work shed light on one possible degradation mechanism of AlGaN-based UVC LEDs.

## Data Availability

The data presented in this study are available on request from the corresponding author.
